# Natural Pigments of Anthocyanin and Betalain for Coloring Soy-Based Yogurt Alternative

**DOI:** 10.3390/foods9060771

**Published:** 2020-06-11

**Authors:** Sandra Dias, Elisabete M. S. Castanheira, A. Gil Fortes, David M. Pereira, M. Sameiro T. Gonçalves

**Affiliations:** 1 Centre of Chemistry, University of Minho, Campus of Gualtar, 4710-057 Braga, Portugal; sandraisdias@hotmail.com (S.D.); gilf@quimica.uminho.pt (A.G.F.); 2 Centre of Physics, University of Minho, Campus of Gualtar, 4710-057 Braga, Portugal; ecoutinho@fisica.uminho.pt; 3 REQUIMTE/LAQV, Laboratory of Pharmacognosy, Department of Chemistry, Faculty of Pharmacy, University of Porto, R. Jorge Viterbo Ferreira, 228, 4050-313 Porto, Portugal; dpereira@ff.up.pt

**Keywords:** soy-based yogurt alternative, betalain pigments, anthocyanin pigments, functional food, food dyes

## Abstract

The aim of this work was to evaluate the color stability of betalain- and anthocyanin-rich extracts in yogurt-like fermented soy, in order to develop a preliminary understanding of how these pigments behave in this type of food system during storage for 21 days at 4 °C. Thus, the extracts of red beetroot, opuntia, hibiscus and red radish were integrated into the yogurt-like fermented soy in two different ways—directly after lyophilization, and encapsulated in nanosystems based in soybean lecithin—as this approach has never been used to further increase the value and potential of the dairy-free alternatives of yogurt-like fermented soy. The results showed that non-encapsulated betalain-rich extracts from red radish are the most promising for coloring yogurt-like fermented soy. However, encapsulated opuntia extracts can also be an alternative to supplement the soy fermented beverages with betalains, without changing significantly the color of the system but giving all its health benefits, due to the protection of the pigments by nanoencapsulation.

## 1. Introduction

The increased consciousness about the impacts of production and consumption of food on environmental, health and ethical issues has motivated consumers to increase their interest in replacing animal-based foods in their diets. Consequently, the food industry has developed new products from plant-based ingredients, that imitate many of the physicochemical and sensory characteristics associated with animal-derived foods, such as fish, meat, eggs, milk and their derivatives [1,2,3]. Plant-based yogurt alternatives are among the most important dairy-free options [4], meeting the abovementioned requirements of consumers, specifically those associated with lactose intolerance and other dairy allergies [5]. In recent years, among the plant-based sources used for the production of plant-based yogurt alternatives, the soybean stands out due to the quantity and quality of its protein, as well as its functional properties [6]. Issues related to texture properties and general appearance are the major challenges faced by producers of plant-based yogurt alternatives.

It is well-known that plant-derived natural pigments, such as betalains, have become popular for use as natural colorants in the food industry [7,8]. Besides, they present pharmacological properties, such as antioxidant, anti-cancer, anti-lipidemic and antimicrobial activities [9,10]. Beetroot (*Beta vulgaris* L.) [11,12,13,14,15,16,17] and opuntia (*Opuntia stricta*) [18,19,20,21,22,23,24,25,26,27], natural sources of betalains, possess the betacyanins isobetanine and betanine, along with betaxanthins, such as indicaxanthin, vulgaxanthin I and vulgaxanthin II (beetroot), as well as other derivatives (opuntia). Moreover, anthocyanins, another important class of natural pigments, have been associated with the delay of the start of diseases related to chronic age, owing to their antioxidant activities, and they also restrain the proliferation of cancer cells as a result of their antiproliferative capacities [28,29,30]. Hibiscus (*Roselle sabdariffa* L.) [31,32,33,34] and radish (*Raphanus sativus* L.) are two sources of anthocyanins that have also been used as colorants of food and beverages, taking advantage not only of their colorimetric potential, but also of their medicinal properties, and they thus have enormous benefits for health [35,36].

In fact, nowadays, interest in the use of betalains and anthocyanins pigments goes far beyond the color they give to food, which, although very important for the visual aspect, is largely surpassed by the health benefits that they can confer, thus enhancing the global value of the products that consumers increasingly want to be functional foods. However, the instability of extracted betalains and anthocyanins may be a weak point that cannot be forgotten, and encapsulation of these pigments in polysaccharide–proteins, gelatin–maltodextrin, carrageenan and liposomes, has emerged as an interesting possible means of minimizing this weakness [15,16,25,26,27].

Keeping all this in mind, the aim of the present study was to evaluate the color stability of betalain- and anthocyanin-rich extracts in a soybean dairy-free alternative, which, in addition to conferring a pleasant pink color, can impart health benefits, further enhancing the final value of the product. Thus, red beetroot, opuntia, hibiscus and red radish were used as sources of betalain- and anthocyanin-rich extracts, and yogurt-like fermented soy was chosen as a soy-based dairy-free model. The additions of the extracts were to already-prepared yogurt-like fermented soy, which prevented the pigments from being exposed to inherent issues during the production, such as temperature, which can affect their stability. However, nanoencapsulation studies were performed with the purpose of evaluating the color obtained in yogurt-like fermented soy as well as its stability, in comparison with those qualities obtained with non-encapsulated pigments, during 21 days of storage. The results obtained point to a promising use of red radish for coloring this type of food. However, encapsulated opuntia extracts can be an alternative for supplementing the yogurt-like fermented soy with betalains, without changing significantly the color of the system, but whilst still giving all its health benefits, due to the protection of the pigments by nanoencapsulation.

## 2. Materials and Methods

### 2.1. Chemicals and Reagents

Ethanol, isopropanol, DMSO, tetrahydrofuran and acetic acid were products from Merck KGaA (Darmstadt, Germany). 3-(4,5-Dimethylthiazolyl-2)-2,5-diphenyltetrazolium bromide (MTT) and Trypan blue were purchased from Sigma-Aldrich (St. Louis, MO, USA). Dulbecco’s Modified Eagle Medium (DMEM), Hank’s balanced salt solution (HBSS), foetal bovine serum (FBS), penicillin–streptomycin solution (penicillin 5000 units/mL and streptomycin 5000 μg/mL) and 0.25% trypsin-EDTA were purchased from GIBCO, Invitrogen™ (Grand Island, NY, USA).

The yogurt-like fermented soy (supplier and distributor—Alpro), with Product code (EAN) 5411188104971, contained the following composition according to the label information: water, shelled soybeans (7.9%), sugar, tricalcium citrate, stabilizer (pectin), acidity regulators (sodium citrate, citric acid), aroma, sea salt, antioxidants (extract rich in tocopherols, esters of acids fat from ascorbic acid), vitamins (B12, D2), live yeast (*S. thermophilus*, *L. bulgaricus*).

### 2.2. Plant Material

The red beetroot, hibiscus and red radish were obtained from a local grocery store in Portugal as fresh materials (red beetroot and red radish), or in dehydrated form (hibiscus), in October 2019. The fresh opuntia was provided by the Opuntia Farm (Albarrol, Portalegre, Portugal), in May 2019.

### 2.3. Preparation of Betalain-Rich Extracts

The red beetroot and opuntia were hand-peeled and their interiors were cut into small pieces. To these small pieces (5 g) of red beetroot or opuntia, a solution of water/ethanol/acetic acid (66.6:33:0.33, *v*/*v*/*v*) (10 mL) was added, and the mixtures were maintained at room temperature, with irradiation protection for 48 h (red beetroot) or 20 min (opuntia) [37]. Then, the mixtures were filtered and the filtrates subjected to centrifugation in a Z 300 universal centrifuge (Hermle LaborTechnik GmbH, Wehingen, Germany) at 500 rpm for 16 min (in case of opuntia), and solvents were partially evaporated under reduced pressure using the rotary evaporator (Rotavapor^®^ R-210, BÜCHI Labortechnik AG, Flawil, Switzerland) at a temperature of 40 °C. At that point, the extracts were subjected to freezing at −80 °C and lyophilization (Alpha 1-4 LD Plus—Christ freeze dryer, Martin Christ Gefriertrocknungsanlagen GmbH, Osterode am Harz, Germany) for five days to produce the corresponding dry extracts which were put in storage in a desiccator until use.

### 2.4. Preparation of Anthocyanin-Rich Extracts

To the hibiscus, in the form it was bought (5 g), a solution of water/ethanol/acetic acid (70:29.7:0.3 *v*/*v*/*v*) (10 mL) was added, and the resulting mixture was maintained with irradiation protection at low temperature (~4 °C), for 72 h [38]. Then, the mixture was filtered, and the solvent was subject to partial evaporation under reduced pressure using the rotary evaporator (Rotavapor^®^ R-210, BÜCHI Labortechnik AG, Flawil, Switzerland) at 40 °C. At that point, the extract was subjected to freezing at −80 °C and lyophilization (Alpha 1-4 LD Plus—Christ freeze dryer, Martin Christ Gefriertrocknungsanlagen GmbH, Osterode am Harz, Germany) over five days, to produce the corresponding dry extract, which was put in storage in a desiccator until use.

The red radish was hand-peeled and the peels were cut into small pieces. To these small pieces (25 g), a solution of water/acetic acid (95:5, *v*/*v*) (100 mL) was added, and the mixture was kept at low temperature (~4 °C) for 18 h. After that, the mixture was filtered, the solvent was partially evaporated, and the resulting extract was subjected to freezing at −80 °C followed by lyophilization, according to a similar procedure as described above for hibiscus [39].

### 2.5. Determination of Betalains Content

The betalains content of red beetroot extract was previously determined [40]. Ultraviolet –visible (UV-Vis) absorption spectra of opuntia extracts were obtained on a Shimadzu UV-Vis-NIR 3600 Plus spectrophotometer (Shimadzu Corporation, Kyoto, Japan). The experimental spectra were decomposed and fitted to a sum of betacyanins and betaxanthins spectra, to determine the concentration of each type of dye, expressed as mg equivalent betanin/L and mg equivalent indicaxanthin/L, as previously described [40]. Betacyanins were detected at maximum wavelength of 538 nm and betaxanthins at λ_max_ = 480 nm, using Equation (1):

Dye content (mg/L) = (A × DF × MW × 1000)/ε × *l* (1)

where A is the absorbance, DF is the dilution factor, *l* is the optical path (1 cm), MW is the molecular weight and *ε* is the molar absorptivity.

In Equation (1), values of *ε* = 6.5 × 10^4^ M^−1^ cm^−1^ and MW = 550 g/mol were used for betacyanin. For betaxanthins, reported values of *ε* = 4.8 × 10^4^ M^−1^ cm^−1^ and MW = 308 g/mol were utilized [41,42].

### 2.6. LRMS and UHPLC-ESI-Q-TOF-MS Analysis of Extracts

Low resolution mass spectrometry (LRMS) analyses were carried out on a Thermo Scientific™ LTQ XL™ linear ion trap mass spectrometer (Thermo Fisher Scientific, Waltham, MA, USA). Ultra-high performance liquid chromatography with electrospray ionization, coupled to quadrupole-time-of-flight mass spectrometry (UHPLC-ESI-Q-TOF-MS) in positive mode (timsTOF) analyses were performed at the “Unidad de Espetrometria de Massa e Proteómica, CACTUS”, at University of Santiago de Compostela, Spain. For the UHPLC, a C18 column was used.

### 2.7. Human Gastric Adenocarcinoma Cell Line Assays

The human gastric adenocarcinoma cell line (AGS) was used as a model for gastric tissue. Cells (Sigma-Aldrich, St. Louis, MO, USA) were maintained in DMEM + GlutaMAxTM-1 with 1% penicillin/streptomycin and 10% FBS, at 37 °C, in a humidified atmosphere of 5% CO_2_.

For assessing viability, cells were plated at a density of 1.5 × 10^4^ cells/well, followed by incubation for 24 h with the samples. After incubation, 0.5 mg/mL MTT solution was added and further incubated for 2 h. The formazan in each well was dissolved in a solution of DMSO/isopropanol (3:1). Lastly, the absorbance at 560 nm was read in a Thermo Scientific™ Multiskan™ GO microplate reader (MA, USA). Viability was expressed as a percentage of the signal of control cells (cell medium).

### 2.8. Nanoencapsulation Studies

The lyophilized extracts of red beetroot, opuntia, hibiscus and red radish were used for nanoencapsulation assays. Ultrapure water Milli-Q grade (MilliporeSigma, St. Louis, MO, USA) was used in all preparation steps. Dilutions in water of the extracts in the range of concentrations 2 × 10^−5^ –50 × 10^−4^ mg/mL were performed to determine the calibration curves and calculate the encapsulation efficiency. The calibration curves are linear in this range of extract concentrations (*R*^2^ = 0.99).

Liposomes were prepared by the ethanolic injection and thin film hydration methods. For the lipid formulation, a commercial lipid mixture used in the food industry was employed, soybean lecithin (Sternchemie, Hamburg, Germany), the composition of which is (% mol/mol) 22% phosphatidylcholine, 20% phosphatidylethanolamine, 20% phosphatidylinositol and 10% phosphatidic acid as main components, with a concentration of 1 × 10^−3^ M. In the ethanolic injection method, 2 × 10^−3^ g/mL extract was added to the lipid mixture, and the solvent was evaporated with a stream of ultrapure nitrogen. Then, tetrahydrofuran (0.75 µL) and ethanol (0.75 µL) were added. The mixture was added to Milli-Q grade water (3 mL) under vortexing and the resulting solution was transferred to Amicon tubes and centrifuged (Universal 320 Hettich Zentrifugen, New Delhi, India), for 10 min at 3000 rpm.

In the case of the thin film hydration method, soybean lecithin (60 mg) was dissolved in ethanol (3 mL), followed by evaporation under an ultrapure nitrogen stream. Then, 10 × 10^−3^ g/mL of the aqueous extract solution, previously dissolved in 5 mL of ultrapure water, was added to the lipid mixture. The solution was vortexed for 5 min and then sonicated for the same time. Finally, the resulting mixture was centrifuged in Amicon filter tubes at 3000 rpm for 10 min.

The encapsulated and non-encapsulated fractions were both collected, and absorption spectra were measured in a spectrophotometer Shimadzu UV-3600 Plus UV-Vis-NIR. The absorbance of these fractions was determined, allowing us to estimate the concentrations of encapsulated and non-encapsulated pigments, using the calibration curve (absorbance vs. concentration) previously obtained. The encapsulation efficiency, EE(%), was calculated using the relation: EE(%) = (Total amount − Amount of nonencapsulated extract)/(Total amount) × 100.

### 2.9. Application of Betalain- and Anthocyanin-Rich Extracts to Soy-Based Yogurt Alternative

In a transparent Petri dish (5 cm^2^), at room temperature, to the commercial yogurt-like fermented soy (Alpro) (2 mL), non-encapsulate extracts of red beetroot (4.5 mg), opuntia (4 mg), hibiscus (15.5 mg), red radish (32.5 mg), or encapsulated extracts of the same species (mass indicated in Table S1 in Supplementary Materials) were added by stirring with glass stirring rod. These mixtures were kept in the refrigerator (at a temperature of 4 °C) for 21 days, and the color was evaluated visually and using the colorimeter at known time periods (after one day, 7 days, 14 days and 21 days of storage).

### 2.10. Color Measurement

The CIELab parameters, L* (brightness), a* (green versus red coordinate) and b* (blue versus yellow coordinate), were measured on a white calibration block using a Chroma Meter CR-400/410 colorimeter (Konica Minolta, Tokyo, Japan).

The yogurt-like fermented soy samples were prepared without or with red beetroot, opuntia, hibiscus or red radish non-encapsulated or encapsulated extracts as mentioned above, and color measurements were made in the transparent Petri dish (5 cm^2^) where they were prepared. Each sample was analyzed at five distinct points.

### 2.11. Statistical Analysis

For biological assays, results were subjected to the Shapiro–Wilk’s normality test to ensure that it followed a normal distribution. Comparison between the means of controls and each experimental condition was performed using ANOVA. The Grubbs’ test was used to identify outliers. Data was expressed as the mean ± standard error of the mean of 3 independent experiments, each performed in triplicate. GraphPad Prism software was used and values were considered statistically significant with a *p* ≤ 0.05.

## 3. Results and Discussion

### 3.1. Betalain- and Anthocyanin-Rich Extracts

In order to obtain betalain- and anthocyanin-rich extracts that can be used in food products, namely dairy-free alternatives such as yogurt-like fermented soy, red beetroot, opuntia, hibiscus and red radish were selected as vegetable sources. After being obtaining, each species was subjected to preliminary studies, in which different experimental parameters were varied, namely the solvent, temperature and time, taking into account the optimization of the pigment extraction process (data not shown). However, for all species only the procedures that resulted in the best results are described, supported by previous published works, which are cited in each case. Thus, starting from red beetroot or opuntia, and using a solution of water/ethanol/acetic acid (66.6:33:0.33, *v*/*v*/*v*) at room temperature, with irradiation protection for 48 h or 20 min, respectively, followed by the usual procedures to separate the filtrates and remove the solvents, it was possible to obtain the corresponding lyophilized betalain-rich extracts [37]. Similarly, starting from hibiscus or red radish, by using water/ethanol/acetic acid (70:29.7:0.3, *v*/*v*/*v*) (hibiscus) [38] or water/acetic acid (95:5, *v*/*v*) (red radish) [39], and keeping them at a low temperature (4 °C) for 18 h (red radish) or 72 h (hibiscus), the lyophilized anthocyanin-rich extracts were obtained. UV-Vis absorption spectroscopy, LRMS and UHPLC-ESI-Q-TOF-MS were used for characterization of the extracts.

**Figure 1:**
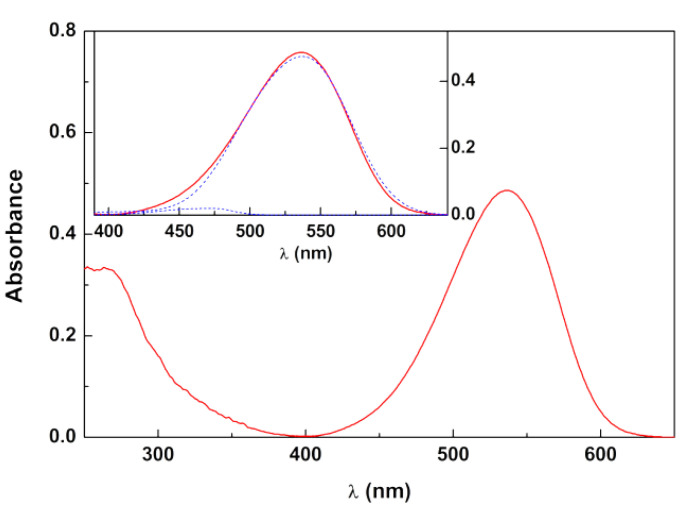
Absorption spectrum of opuntia extract in aqueous solution (at a concentration of 4 mg/mL). Inset: Spectral decomposition in the visible spectral region to obtain betacyanins and betaxanthins concentrations.

Based on UV-Vis absorption spectra of aqueous solutions of the lyophilized extracts, the anthocyanin content was previously estimated for red radish and hibiscus, together with the betalain content for red beetroot [40]. Regarding the new opuntia extract, the UV-Vis spectrum was fitted to a sum of the betacyanins spectrum (maximum at λ = 538 nm) and betaxanthins spectrum (maximum at 480 nm) (Figure 1, Table 1), as these classes of compounds have characteristic and distinguishable absorption spectra [43]. Using the reported spectra for each of the components [43], the content of each dye was determined by Equation (1), and expressed as mg equivalent betanin/L and mg equivalent indicaxanthin/L, respectively [44,45]. A very low content of betaxanthins in the opuntia extract could be observed, betacyanins being the dominant class of dyes in this plant extract.

**Figure 2:**
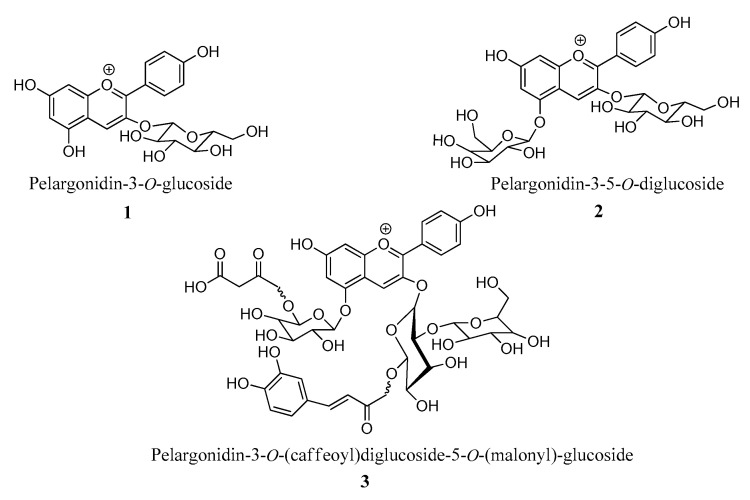
Structures of pelargonidin derivatives **1**–**3** putatively present in red radish extract.

Red beetroot extract, analyzed via LRMS acquired in a positive ionization mode (ESI), displayed a peak with m/z 550.78 (base peak), attributed to the betanin pigment, according to the literature [14,40]. In the case of red radish extract, UHPLC ESI-Q-TOF-MS/MS analyses revealed the presence of 16 compounds, some of which showed molecular mass compatible with pelargonidin derivatives, possibly acylated 3-5-heterosides (Table 2). Figure 2 (1–3) shows the structure of some pelargonin derivatives, with molecular masses compatible with analytical data.

**Figure 3:**
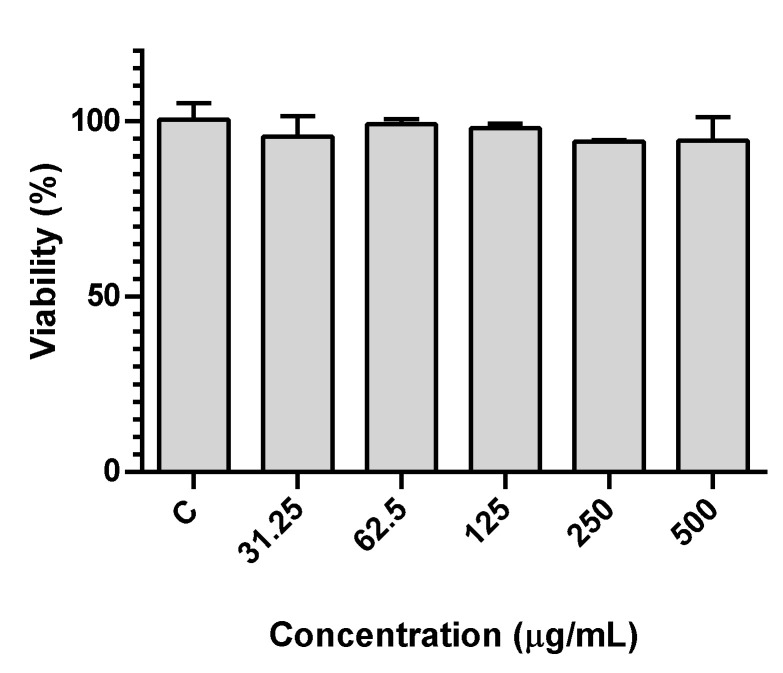
Viability of AGS cells exposed to opuntia extract for 24 h, as assessed by the MTT assay.

Given the intended application in yogurt-like fermented soy, as well as other potential applications in food, the cytotoxicity of the lyophilized extracts in human gastric adenocarcinoma (AGS) cells, as a model for gastric cells, was evaluated after exposure for 24 h. We have previously described that the extracts of red beetroot, hibiscus and red radish had no significant impact on cell viability in this in vitro model [40]. We now describe that the same is true for the opuntia extract, in which no loss of viability was detected up to 0.5 mg/mL (Figure 3), which points to the lack of toxicity of all extracts.

### 3.2. Application of the Extracts to Soy-Based Yogurt Alternative

In order to evaluate the stability of the color of betalain- and anthocyanin-rich extracts from red beetroot, opuntia, hibiscus and red radish, in alternative dairy-free products, such as soy-based products, a yogurt-like fermented soy was chosen as the food model. The extracts were applied in both non-encapsulated form and encapsulated in liposomes of soybean lecithin, as these systems are reported as useful in the nanoencapsulation/stabilization of different components, as well as being additives in the food industry [45]. The ethanolic injection and thin film hydration methods were used, as these are usually associated with high encapsulation efficiencies, depending on the hydrophilicity or hydrophobicity of the extracts [46,47].

The extracts were added to already-prepared yogurt-like fermented soy, preventing the pigments from being exposed to inherent issues during the production, such as temperature and pH, which can affect their stability. After addition, the food systems were kept at a low temperature (~4 °C) for 21 days of storage.

**Figure 4:**
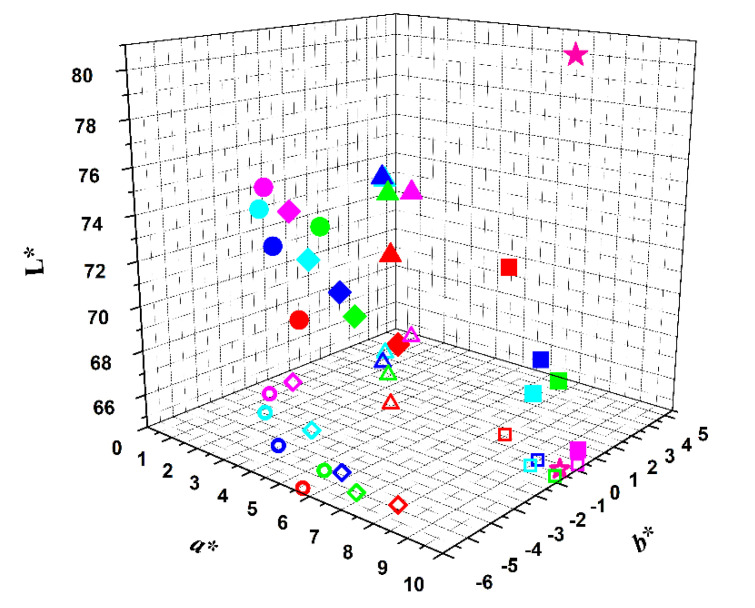
Representation of the colors of the several samples in L*a*b* color scheme, immediately after extract addition (red symbols), and after 1 day (green symbols), 7 days (blue symbols), 14 days (cyan symbols) and 21 days (magenta symbols) of storage at 4 °C. Projection in a*,b* plane: open symbols. 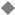: red beetroot; 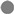: opuntia; 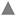: hibiscus; 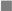: red radish;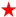: commercial strawberry flavor yogurt.

The color obtained in the yogurt-like fermented soy samples was evaluated visually and by measuring color indexes in a colorimeter, where L* is the degree of lightness, covering a range from black (0) to white (100), a* is the degree of redness and greenness (from −80 to 0 = green; from 0 to 100 = red), and b* is the degree of yellowness and blueness (from −100 to 0 = blue; from 0 to +70 = yellow). Table S1 in the Supplementary Material shows the results obtained after the addition of the extracts (0 days) at regular time periods, namely on the 1st, 7th, 14th and 21st days of storage (Figure 4), in comparison with the yogurt-like fermented soy without the addition of any extract. The color of a commercial strawberry flavored yogurt was also analyzed (L* = 81.19; a* = 9.66; b* = −0.24), and used for comparison. It can be observed, by the projection in the color plane (a*,b*),that the most stable colors, in the whole storage period of 21 days at 4 °C (monitored at 7 days intervals), are the ones obtained from hibiscus and from red radish, especially the latter. Further, the yogurt-like fermented soy with the red radish pigment, despite the lower L* value, is the most similar in color to the commercial sample (Figure 4).

**Figure 5:**
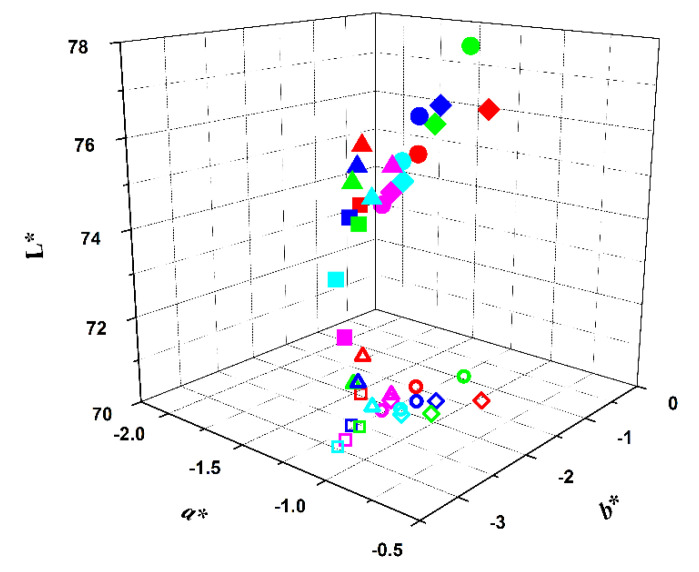
Representation of the colors of the several samples in L*a*b* color scheme immediately after extract addition (encapsulated in soybean lecithin liposomes by ethanolic injection) (red symbols), and after 1 day (green symbols), 7 days (blue symbols), 14 days (cyan symbols) and 21 days (magenta symbols) of storage at 4 °C. Projection in a*,b* plane: open symbols. 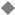: red beetroot; 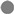: opuntia; 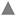: hibiscus; 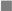: red radish.

**Figure 6:**
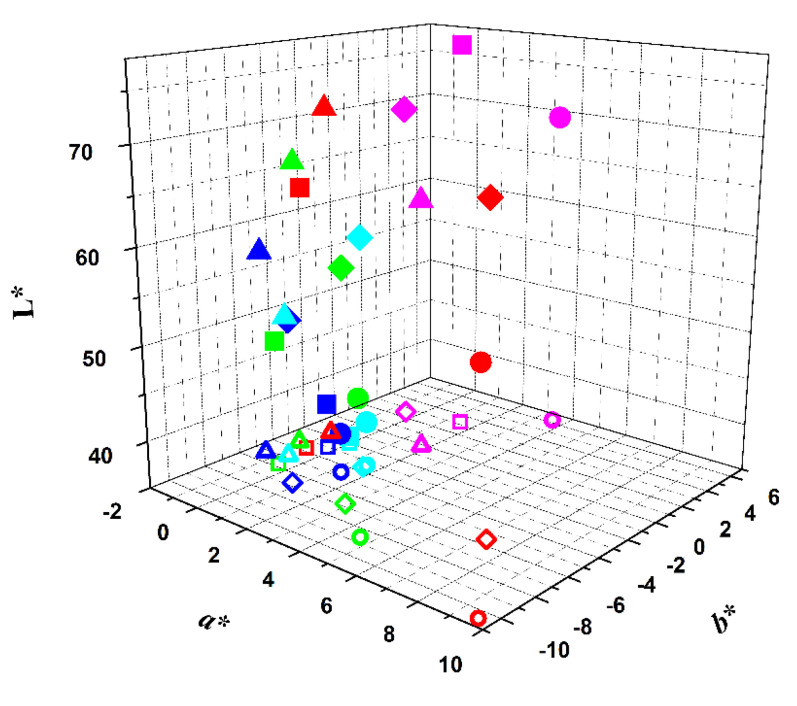
Representation of the colors of the several samples in the L*a*b* color scheme immediately after extract addition (encapsulated in soybean lecithin liposomes by thin film hydration), (red symbols), and after 1 day (green symbols), 7 days (blue symbols), 14 days (cyan symbols) and 21 days (magenta symbols) of storage at 4 °C. Projection in a*,b* plane: open symbols. 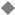: red beetroot; 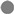: opuntia; 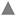: hibiscus; 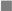: red radish.

The same study was performed with encapsulated extracts in soybean lecithin liposomes, prepared either by ethanolic injection method or thin film hydration (Table S1 and Figure 5 and Figure 6). Both methods have shown very high encapsulation efficiencies for extracts of beetroot, red radish and hibiscus [40]. For the opuntia extract, high encapsulation efficiencies were also obtained (Table 3), although these were slightly lower for the ethanolic injection method. Nevertheless, it was observed that the thin film hydration method needs high quantities of the extract to obtain a visually appealing pink color, by comparison with the commercial yogurt.

It can be concluded that the encapsulation by ethanolic injection method (Figure 5) protects the dyes, but also changes the color, as the encapsulation systems are yellowish due to the soybean lecithin components. However, this is not very significant, as the b* values are slightly negative, but so are the a* values. These results suggest that the ethanolic injection method can be an interesting alternative when the objective is to complement the food system with betalain or anthocyanin pigments, without causing a significant change in its color.

Regarding the thin film hydration method (Figure 6), the color was not stable for the period of 21 days, with significant and erroneous changes in color parameters and lightness, indicating a non-protection of the dyes or an uncontrolled (non-gradual) release from the nanocarriers.

In order to obtain a visually satisfactory and interesting pink color comparable with the commercial sample, the yogurt-like fermented soy samples must have positive a* values (which suggest the presence of red pigments), and b* values close to zero, as yellow or blue pigments must not contribute significantly to the resulting color. The data obtained with non-encapsulated extracts show that on day 0, the highest value of a* occurred with red beetroot extract (a* = 7.84), followed by the red radish, opuntia and hibiscus (a* = 3.85) extracts, in that order. Further, on day 0, the highest b* index was displayed by red radish extract (b* = 0.61), followed by hibiscus, red beetroot and opuntia (b* = −5.78). Over the 21-days storage period, it appears that the values of a* decreased in all samples with non-encapsulated extracts, except for the sample with red radish, which increased (a* = 9.86). As for the b* values, over this period, all samples approached zero, except in the case of the hibiscus extract that increased to a positive value (b* = 5.24), thus registering the contribution of yellow pigments to its color. The visual observation of the samples cohered with the results of the colorimeter, that is, the most stable color was given by the red radish extract. These results may be due to the high amount of pigment added in the case of the radish extract, and its stabilizing properties (copigmentation) [48]. The color obtained with the remaining extracts was lost over time, probably due to the instability of the pigments in the yogurt-like fermented soy at its usual pH (pH value is 4.8) [49,50,51]. In fact, decreases in pH, a function of the lactic acid bacteria in fermented milk, cause an increase of the hydroxylation of the phenolic rings, which could result in a change of pigments towards blueish tones, as previously observed by other authors for anthocyanin-based extracts [52,53].

Regarding the samples of yogurt-like fermented soy with extracts encapsulated by the ethanol injection method, on day 0, the highest a* value was shown by the sample with red beetroot extract (a* = −0.98), followed by the one with opuntia, red radish and hibiscus (a* = −1.78). On the 21st day, the best result appeared in the sample with the red radish extract (a* = −1.19). Considering b* values, on day 0, the most suitable values were obtained with red beetroot (b* = −1.31) and hibiscus (b* = −1.21) extracts (with very close values), and over the 21 days, the extracts in the yogurt-like fermented soy with the best b* values remain unaltered (b* = −1.89, red beetroot; b* = −1.78, hibiscus). The lower values of a* and b* in the first day of the experiment are due to the fact that the pigments are encapsulated in soy lecithin. It would be expected that over time these values would improve, as a result of the release of the pigments. However, there is only a slight increase, with the exception of red beetroot, which suggests that red radish, hibiscus and opuntia extracts are protected by nanoencapsulation, and after their release, there is no pigment degradation. As for red beetroot, the decrease in the values of a* and b* may indicate that the encapsulation did not protect the dyes in the yogurt-like fermented soy sample, and the pigments suffered degradation, probably due to the low pH of the yogurt-like fermented soy.

In the samples of yogurt-like fermented soy with extracts encapsulated by the thin film hydration method, on day 0, the highest a* values were recorded for opuntia (a* = 9.6), followed by red beetroot, red radish and hibiscus (a* = −1.07). Regarding the b*, the best value was obtained with hibiscus extract (b* = −1.65), followed by red radish, red beetroot and opuntia (b* = −10.51). Over the 21 days of storage, the sample with opuntia extract continued to display the highest a* value (a* = 3.52), followed by hibiscus, red radish and beetroot (a* = −0.48). The best result for b* was recorded in the hibiscus extract (b* = −0.08). A significant increase in b* over 21 days was registered in the samples with opuntia, red radish and beetroot extract, but this did not occur with the hibiscus extracts. Further, as mentioned, this method needed a high amount of the extracts in order to obtain a satisfactory color.

Overall, considering the results with the encapsulated extracts, it seems that protection of the pigments occurred in the cases of red radish and hibiscus extracts, since there was an increase in both their a* and b* indexes. On the other hand, the application of opuntia and beetroot extracts resulted in a decrease of a* and b* values, which indicates a decrease in the stability of the pigments after their release. Therefore, with regards to application in yogurt-like fermented soy, encapsulation may be considered if there is a need to protect the dyes from degradation, and, for this purpose, a further investigation is needed regarding lipid composition and stability.

## 4. Conclusions

Betalain-rich extracts of red beetroot and opuntia, as well as anthocyanin-rich extracts of hibiscus and red radish, were obtained. Incorporation of the extracts in soybean lecithin nanosystems was performed via ethanolic injection and film hydration, which resulted in moderate to excellent encapsulation efficiencies. Non-encapsulated and encapsulated extracts were added to yogurt-like fermented soy, and color stability was visually and colorimetrically evaluated during storage at ~4 °C, for 21 days. The results showed that the non-encapsulated extract of red radish afforded to yogurt-like fermented soy the most appealing pink color, which suggested that this extract may be very promising for future coloring applications in soybean-based food products. On the other hand, nanoencapsulated opuntia extracts can also be used to supplement yogurt-like fermented soy with betalains, imparting their health benefits without changing significantly the color.

## Figures and Tables

**Table 1:** Concentration of pigments in the extracts used determined by UV-Vis absorption.

Extract	Betalains (mg/L)		Anthocyanins (g/L)
	Betacyanins	Betaxanthins	
Red Beetroot	20.1 ^1^	4.27 ^1^	-
Opuntia	4.10	0.13	-
Hibiscus	-	-	13.01 ^1^
Red radish	-	-	1.27 ^1^

^1^ Previously determined [40].

**Table 2:** Mass spectrometry data and putative identification for red radish extract based on the known molecular weight and observed M^+^.

Entry	M^+^	Compound
1	403	-
2	271	Pelargonidin
3	433	Pelargonidin-3-*O*-glucoside
4	1181	Pelargonidin-3-*O*-(caffeoyl, feruloyl)-diglucoside-5-*O*-(malonyl)-glucoside
5	989	Pelargonidin-3-*O*-(*p*-coumaroyl)diglucoside-5-*O*-(malonyl)-glucoside
6	1005	Pelargonidin-3-*O*-(caffeoyl)diglucoside-5-*O*-glucoside
7	1019	Pelargonidin-3-*O*-(feruloyl)sophoroside-5-*O*-glucoside
8	1135	Pelargonidin-(*p*-coumaroyl,*p*-coumaroyl)-diglucoside-5-*O*-(malonyl)-glucoside
9	1165	Pelargonidin-3-*O*-(feruloyl,*p*-coumaroyl)-diglucoside-5-*O*-(malonyl)-glucoside

**Table 3:** Encapsulation efficiencies, EE (%) ± SD (%), of the several plant extracts in soybean lecithin liposomes, prepared by either by ethanolic injection or thin film hydration (SD: standard deviation).

Species	Ethanolic Injection	Thin Film Hydration
Red Beetroot	98.4 ± 3.1 ^1^	99.3 ± 3.6 ^1^
Opuntia	90.9 ± 1.8	92.8 ± 2.0
Hibiscus	99.2 ± 4.8 ^1^	98.1 ± 2.9 ^1^
Red radish	99.9 ± 6.2 ^1^	99.6 ± 4.2 ^1^

^1^ Previously determined [40].

## Supplementary Materials

The following are available online at https://www.mdpi.com/2304-8158/9/6/771/s1, Table S1: Variations in color index of soy-based yogurt alternative during 21 days of storage. Soy-based yogurt alternative – SY; B – Red beetroot; O – Opuntia; H - Hibicus; R - Red radish; Ei - Ethanolic injection method; Fh - thin film hydration method. Amounts of extracts (mg) are given in brackets.
