# Supplementary material for: Natural Pigments of Anthocyanin and Betalain for Coloring Soy-Based Yogurt Alternative
Source: Foods. 2020 Jun 11;9(6):771. doi: 10.3390/foods9060771 (PMC7353562; doi:10.3390/foods9060771)
Supplement: Supplementary file 1 [file foods-09-00771-s001.pdf]

Supplementary Material

Natural Pigments of Anthocyanin and Betalain for Coloring Soy-Based Yogurt Alternative

Sandra Dias [1], Elisabete M. S. Castanheira [2], A. Gil Fortes [1], David M. Pereira [3] and M. Sameiro T. Gonçalves [1]*

[1] Centre of Chemistry, University of Minho, Campus of Gualtar, 4710-057 Braga, Portugal; sandraisdias@hotmail.com (S.D.); gilf@quimica.uminho.pt (A.G.F.)
[2] Centre of Physics, University of Minho, Campus of Gualtar, 4710-057 Braga, Portugal; ecoutinho@fisica.uminho.pt
[3] REQUIMTE/LAQV, Laboratory of Pharmacognosy, Department of Chemistry, Faculty of Pharmacy, University of Porto, R. Jorge Viterbo Ferreira, 228, 4050-313 Porto, Portugal; dpereira@ff.up.pt
* Correspondence: msameiro@quimica.uminho.pt; Tel.: +351253604372

Received: date; Accepted: date; Published: date

Table S1. Variations in color index of soy-based yogurt alternative during 21 days of storage. Soy-based yogurt alternative – SY; B – Red beetroot; O – Opuntia; H - Hibicus; R - Red radish; Ei - Ethanolic injection method; Fh - thin film hydration method. Amounts of extracts (mg) are given in brackets.

Sample	Lab index during storage														
	**0 day**			**1st day**			**7th day**			**14th day**			**21st day**		
	**L***	**a***	**b***	**L***	**a***	**b***	**L***	**a***	**b***	**L***	**a***	**b***	**L***	**a***	**b***
SY	80.98	-1.87	2.35	83.10	-1.61	3.76	81.15	-1.64	1.31	81.31	-1.24	1.41	79.88	-1.15	0.56
SY+B (4.5)	70.98	7.84	-4.81	71.71	6.70	-5.02	72.09	5.72	-4.40	72.17	3.42	-2.92	72.84	0.71	-0.63
SY+ O (4.0)	71.44	5.58	-5.78	74.65	5.31	-4.62	73.20	3.44	-4.33	73.86	1.68	-2.94	74.31	0.82	-1.81
SY+H (15.5)	71.51	3.85	0.14	73.46	2.41	1.92	73.83	1.62	2.66	73.43	1.15	3.40	72.22	0.84	5.24
SY+R (32.5)	69.43	7.45	0.61	68.57	9.78	-0.68	68.88	8.95	-0.16	67.66	9.01	-0.60	65.17	9.86	0.31
SY+B Ei (7.9)	76.61	-0.98	-1.31	76.43	-1.07	-1.87	76.70	-1.14	-1.61	75.24	-1.17	-2.07	74.82	-1.31	-1.89
SY+O Ei (8.29)	75.48	-1.32	-1.47	77.76	-1.22	-0.94	76.47	-1.21	-1.74	75.60	-1.21	-1.98	74.68	-1.27	-2.12
SY+H Ei (7.91)	75.28	-1.78	-1.21	74.80	-1.58	-1.82	75.15	-1.57	-1.77	74.77	-1.33	-2.12	75.31	-1.35	-1.78
SY+R Ei (7.98)	74.44	-1.47	-1.96	74.99	-1.23	-2.53	74.61	-1.27	-2.56	73.62	-1.17	-2.97	72.27	-1.19	-2.82
SY+B Fh (21.35)	67.52	7.19	-5.54	59.01	2.88	-6.78	52.30	0.76	-6.72	59.69	1.51	-3.56	70.01	-0.48	2.58
SY+O Fh (20.52)	57.50	9.60	-10.51	48.78	4.61	-8.62	39.25	1.29	-4.59	39.81	1.53	-3.33	69.56	3.52	6.33
SY+H Fh (20.85)	71.09	-1.07	-1.65	65.92	-1.28	-3.35	57.16	-1.45	-5.18	50.43	-0.76	-4.78	62.17	1.74	-0.08
SY+R Fh (21.05)	63.62	-0.77	-3.67	48.50	-0.51	-5.74	39.92	-0.30	-3.01	35.77	-0.04	-2.00	77.33	1.42	3.25
